# Supplementary material for: Quinoa (Chenopodium Quinoa Willd.) as Functional Ingredient for the Formulation of Gluten-Free Shortbreads
Source: Foods. 2024 Jan 24;13(3):377. doi: 10.3390/foods13030377 (PMC10855087; doi:10.3390/foods13030377)
Supplement: Supplementary file 1 [file foods-13-00377-s001.zip › foods-2825592-supplementary.pdf]

SHORTBREADS QUANTITATIVE DESCRIPTIVE ANALYSIS SHEET

Panelist's code: ____

Date: ______________________

Sample: ____

You are asked to evaluate shortbreads by quantitative descriptive analysis. You are asked to identify and determine descriptors relating to smell, taste, and texture. The descriptors and their corresponding definitions are available to the panelists during all sessions. A 10-point intensity scale (from 0 to 9) is used.

APPEARANCE

uniformity 0____9

color 0____9

global appearance 0____9

ODOR

sweetness 0____9

vanilla 0____9

toasted 0____9

buttery 0____9

overall odor 0____9

TASTE

bitter 0____9

buttery 0____9

vanilla 0____9

floury 0____9

sweetness 0____9

saltiness 0____9

eggy 0____9

overall taste 0____9

CONSISTENCY

crispness 0____9

hardness 0____9

dryness 0____9

pastiness 0____9

Free comments:

____

____

____

____

____

____

____

____

____

triangle-discriminating test

Panelist's code: ____

Date: _____________________

SAMPLE A **SAMPLE B** **SAMPLE C**

Different sample:	A	B	C
Preferred sample:	A	B	C
